# Two is better than one: Apologies from two robots are preferred

**DOI:** 10.1371/journal.pone.0281604

**Published:** 2023-02-22

**Authors:** Yuka Okada, Mitsuhiko Kimoto, Takamasa Iio, Katsunori Shimohara, Masahiro Shiomi

**Affiliations:** 1 Department of Agent Interaction Design Laboratory, Advanced Telecommunications Research Institute International, Kyoto, Japan; 2 Department of Information Systems Design, Doshisha University, Kyoto, Japan; 3 Faculty of Culture and Information Science, Doshisha University, Kyoto, Japan; Teesside University, UNITED KINGDOM

## Abstract

Although the capabilities of service robots are increasing, avoiding any mistakes is difficult. Therefore, strategies for mitigating mistakes, such as apology behavior designs, are essential for service robots. Past studies reported that costly apology is perceived as more sincere than non-costly ones and more acceptable. To increase the apology cost in robot service situations, we thought that using multiple robots would increase the perceived costs in the of financial, physical, and time costs. Therefore, we focused on the number of robots who apologize for their mistakes as well as their individual, specific roles and behaviors during such apologies. We investigated the differences in perceived impressions toward apologies from two robots (the main robot that makes a mistake and apologizes and a sub-robot that also apologizes) and an apology from just one robot (only the main robot) through a web survey with 168 valid participants. The experiment results showed that the participants significantly preferred and positively evaluated apologies from two robots more than one robot in the context of forgiveness, negative word-of-mouth, trust, and intention to use. We also conducted another web survey with 430 valid participants to investigate the effects of different roles for the sub-robot: apologize-only, cleaning-up-only, and both actions. The experimental results showed that the participants significantly preferred and positively evaluated both actions in the context of forgiveness and reliable/competent perspectives.

## Introduction

Service robots are becoming increasingly common in daily environments. During the COVID-19 pandemic, they conducted various daily activities to mitigate the risk of infection [1, 2]. In the human-robot interaction research field, robotics researchers have investigated the effectiveness of social robots in shopping malls [3–5], public transportation [6–8], science museums [9–11], and nursing homes [12–14]. These studies described what services and systems were useful to support daily living before the pandemic. These long-term academic contributions have fueled recent advances in service robots in commercial contexts.

For such service robots for daily environments, robotics researchers should consider a situation where robots are not perfect. Since we human beings obviously make mistakes, we have several strategies to mitigate them, such as offering an apology as a “social lubricant” [15]. In fact, people prefer to interact with a company that apologizes compared to a company that does not [16]. Several studies investigated the effects of apologies in economic and business contexts [17, 18] because the cost perspective is one key factor for accepting them. A past study has already proved that apology-forgiveness mechanisms emerge if the apology is costly enough up to a certain threshold [17]. Moreover, other studies provided a computational model that can explain the effects between apology’s cost and the degree of commitment, and the relationship between them and the iterated Prosoner’s Dilmma [19, 20]. Another study reported the importance of such costly apologies, which tend to be perceived as more sincere than non-costly ones [18]. A recent study in human-computer interaction reported that a sincere apology increases feelings of forgiveness, perceived intelligence, and likeability toward agents [21]. These studies suggest that the concept of cost in apologies and their sincerity are essential for mitigating strategies for social robots.

Although robotics researchers did not clearly include a cost-like concept, they have already implemented behaviors that diminish the mistakes of service robots and lead to forgiveness [22–29]. However, we note that the existing studies of robot mitigation strategies mainly focused on a situation where a single robot apologizes. In the field of human-robot interaction, several studies have investigated situations where multiple robots collaborate to provide services. Several studies reported the effectiveness of using multiple robots during interactions with people, such as rewarding [30, 31] and information-providing services [32–34]. These studies reported that even though the amount of information is identical between multiple robots and a single robot, people prefer the former.

Based on these considerations, we are interested in whether using multiple robots when apologizing is as effective as other service contexts. We thought that using multiple robots for an apology might increase the perceived costs more than an apology from just one robot in the context of financial, physical, and time costs, creating a situation that leads people to absolve a robot for its failures and has positive impressions about it.

**Fig 1:**
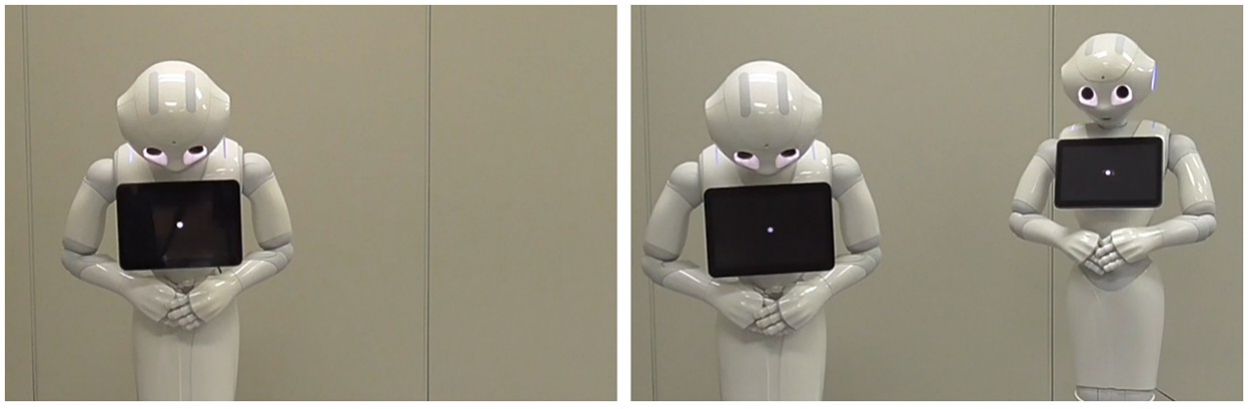
A scene of apology. Apology from one robot (left) and apologies from two (right).

We believe that apologies from multiple robots are a more prudent approach than a single apology from just one robot: e.g., the main robot that apologizes for its mistake (Fig 1, left) and a sub-robot that also apologizes (Fig 1, right)). Investigating the effectiveness of multiple-robot-apologizing behaviors will provide useful knowledge to design such mitigating strategies for service robots in actual environments. Based on these considerations, we address the following research question:

Research question 1: Do people prefer apologies from two robots or just one?

This study also focuses on the sub-robot’s role that supports the main robot. The sub-robot can apologize after the main robot, although it can also fulfill a different role, such as cleaning up spilled food while the main robot apologizes for knocking it over. Investigating the effectiveness of the sub-robot’s behavior will also provide useful knowledge in collaboration with other robots in the context of mitigating failures. Based on these considerations, we pose another research question:

Research question 2: Should the sub-robot only apologize, clean up, or do both?

## Related works

### Apology in human-robot interaction

Similar to apologies in human-human interaction, apologies from robots are useful to diminish failures. Robotics researchers implemented different kinds of apology behaviors for social robots and investigated their effectiveness. A typical approach is to design a verbal apology and offer compensation to mitigate a failure [24, 28] because verbal behavior design is useful for such robots as non-humanoids and simply designed social robots that lack sufficient degree of freedoms.

Non-verbal behavior design is another typical approach to mitigate failures. Researchers focused on reaction behaviors when robots fail to show that the robots understand their own mistakes [25, 26]. Apologies with non-verbal behaviors are limited to social robots with adequate capabilities to express various motions, such as humanoid robots, but integrating both verbal and non-verbal behaviors is probably more effective for mitigating failures [27]. Another study reported the importance of showing remorse in a robot apology, including both verbal and non-verbal behaviors [29]. Such apology behaviors are effective not only in human-robot interaction but also in human-agent interaction contexts [35–38].

Another study, which focused on the appearance of robots in apology situations [39], reported that humanoid service robots can recover more fully than non-humanoid robots from a service failure by giving a sincere apology. This result suggests that the perceived impressions toward robots influence whether people can forgive them.

Although these studies showed the effectiveness of their behavior designs in apology situations, as described above, they mainly focused on how to design acceptable apologies with only one robot. Unlike these past studies, we focused on the effects of using multiple robots for apology situations and evaluated their effectiveness.

### Number effects of social robots

Using multiple robots for services has become a popular approach. For example, researchers developed a multiple-robots-based information-providing system in which two robots discuss specific content, proving that such a style effectively attracts people in real environments [32, 40]. Researchers argued that observations of conversational-style information-providing between robots are easier for people than direct interaction with a social robot. Other studies reported the effectiveness of using multiple robots in education contexts for children [31, 34] and conversational services for the elderly [41]. These past studies described the effects of the behavior and the impression changes of the people involved by interaction with multiple robots.

Although these studies showed the effectiveness of using multiple robots in various situations, they focused less on apology situations. In other words, the effectiveness of multiple robots in apology situations has not yet been investigated. Although a past study in human science focused on costly group apology effects, it did not compare the number of persons who apologized [18]. Our study provides novel knowledge about the effectiveness of using multiple robots and their behavior designs in apology situations.

## Experiment I

Experiment I has two aims: 1) investigating and comparing the effectiveness of apologies from two robots with an apology from just one to answer research question 1; 2) gathering opinions from participants about whether the second robot should only apologize or do other tasks. The second aim led to designing Experiment 2 which answers research question 2.

### Hypothesis and prediction

An apology is an essential action to mitigate failure and be forgiven. Past studies in human science literature showed that the cost of apologies is an important factor in accepting them [16, 17, 42, 43]. Although such cost perspectives are not directly involved, robotics researchers investigated the effectiveness of sincere apology behaviors for service robots that fail in daily environments [24–28].

To create a more acceptable apology for a robot, we focused on the number of robots that apologize. Past studies showed the effectiveness of using multiple robots in service contexts [31, 32, 34, 40, 41]. Moreover, using multiple robots simply increases the perceived cost of apologizing compared to a single robot. However, these studies did not deal with apology situations. Therefore, the effects of apologies from multiple robots are still unknown. Based on these considerations, we hypothesized that multiple robot apologies would have an advantage compared to one robot apology. Following this hypothesis, we made the following prediction:

**Prediction 1**: If multiple robots apologize for their failure, people will accept the apologies and have more positive impressions than one robot’s apology.

### Visual stimuli and conditions

We made videos where a food-service robot drops a customer’s order and apologizes. We used these videos in a web survey because of the obvious difficulties with such enactment in an actual restaurant. We used Pepper, developed by Softbank Robotics, which has 20 degrees of freedom (DOFs), stands 121 cm tall, and weighs 29 kg. Each video’s resolution was 1286 x 762 pixels; the fps was 30, and the lengths were about 27 seconds.

**Fig 2:**
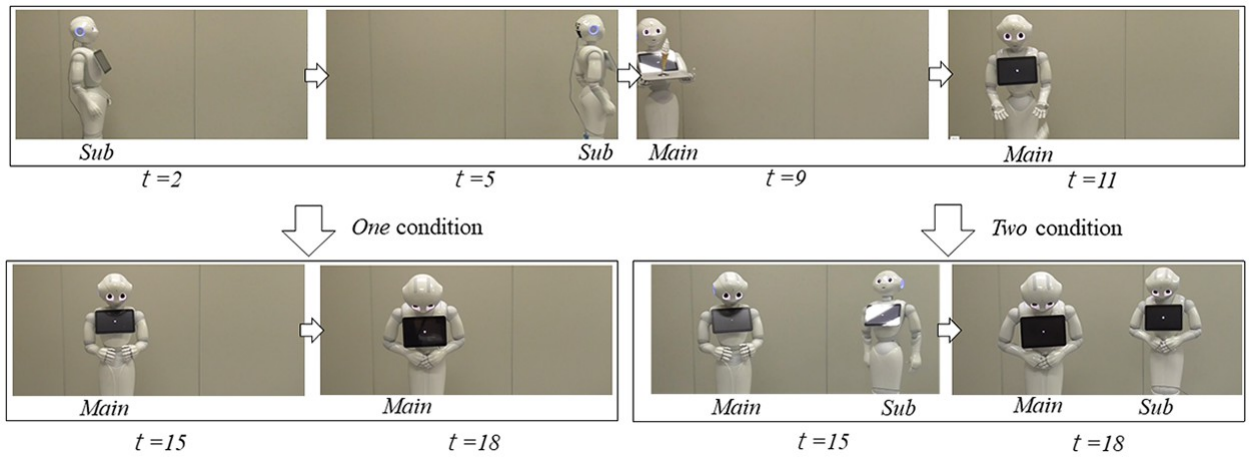
Visual stimuli of Experiment I. Snapshots with specific timings (*t* = seconds).

In this experiment, we prepared one factor (number factor, one/two conditions) and took two videos (Fig 2). In both videos, two robots appear (the main robot serves the food, and the sub-robot is another restaurant worker). First, the sub-robot crosses from left to right to explain that there are two robots in the video stimuli. Next, the main robot delivers an ice cream cone from the left and says, "Here you are." Unfortunately, it drops the cone and says, "Sorry." The main robot apologizes and offers to serve new ice cream without charging for it, and tell that no payment was required toward the original order too. The main robot’s speech contents (apologizing and offering compensation), which are identical between conditions, are based on a past study that investigated appropriate mitigating strategies for robot failures [24].

In the *one*-apology condition, the main robot apologizes, and the sub-robot only appears in the beginning. In the *two*-apologies condition, the sub-robot enters from the right and apologizes in the middle of the main robot’s apologizing. Therefore, the difference between the conditions is whether the sub-robot apologizes following the main robot after the latter’s mistake. We did not prepare a *no*-apology condition because a past study reported the effectiveness of a robot’s apology, i.e., a *one*-apology condition is better than a *no*-apology condition [24]. Therefore, we set the baseline of our study to the *one-*apology condition.

We note that the main robot’s behaviors are completely the same between the two conditions because we used the same programs to control its motions. The detailed behaviors are described in Table 1.

**Table 1:** The timeline of both the main and sub-robot in the *two*-apologies conditions.

t (s)	Main robot	Sub robot
1		Enter from the left
6		Exit from the right
8	Deliver an ice cream	
10	Drop the ice cream	
14		Enter from the right
15	Apologize and bow	
17		Bow
20	Offers to serve new ice cream	
23	Tell that no payment was required	
24	Bow again	
25		Bow again

### Measurement

We prepared a question with three choices to investigate the preferred number of robots during an apology. The participants chose their preferred number of apologizing robots from three choices: one, two, and either one. Having an additional choice as an intermediate option allows us to investigate whether an apology by two robots is effective. If we prepared only two choices (i.e., one or two), the participants are forced to choose even if they feel there is no actual difference.

We also measured questionnaire items to investigate the perceived feelings from various viewpoints based on past studies on the effects of service-recovery strategies and trust (all items are described in the (S1 File)). For the five questionnaires, we employed and modified the following: the forgiveness scale [44], negative word-of-mouth (NWOM) [44], performance trust from the multi-dimensional measure of trust (consisting of reliable and competent subscales) [45], moral trust from the multi-dimensional measure of trust (consisting of ethical, transparent, and benevolent subscales) [45], and intention to use (ITU) [46]. Each questionnaire item of each scale was assessed using a one-to-seven response format, where 1 was the most negative and 7 was the most positive.

We investigated the forgiveness, NWOM, and ITU scales about a shop and the trust scales toward the robot clerk serving the ice cream. We also prepared a free-text form to gather opinions from participants about whether only the second robot should apologize or do another task. The participants freely gave their opinions.

Due to the need for such screening of participants in web surveys [47, 48], we prepared two dummy questions to confirm whether participants carefully watched the videos and instructions at the end of the questionnaires. The first question asks the participants to answer what was dropped out by the robot in the video with four-choice questions. We excluded the participants who wrongly answered. The second question is based on the instruction manipulation check [48]. We translated the text of the example instruction manipulation check (Fig 1 in [48]) to Japanese text and prepared three responsible format items. The text asks participants to skip answers to confirm whether they have read the instruction. Therefore, we also excluded the participants who answered these dummy questions.

### Procedure

All the procedures were approved by the Advanced Telecommunication Research Review Boards (20-501-3). The consent form is displayed in the beginning of the first webpage. The participants who only agreed with it can participate in the survey. First, the participants read explanations of the experiment and how to evaluate each video, and then we verified that they could clearly hear the video’s audio. We also displayed text to support imagining a situation where multiple robots work in a cafeteria.

Next, they observed a video with the *one*-apology or *two*-apologies robots apologizing conditions and answered the questions. They again watched a video with *two*-apologies or *one*-apology conditions and answered the questions. After watching both videos, they answered the three-choice question and completed the free-text form. Finally, they answered the dummy questions to determine how carefully they watched the video and to verify the quality of their answers.

### Participants

The experiment was conducted using the participant pools of a Japanese crowd-sourcing company (CrowdWorks, Inc., which has more than three million registered workers). 203 people (101 women, 101 men, and 1 who declined to specify gender) joined our experiment. The screening process winnowed that number to 168 valid participants: 81 women, 86 men, and 1 who declined to specify.

## Results

### Preferred number of apologizing robots

**Fig 3:**
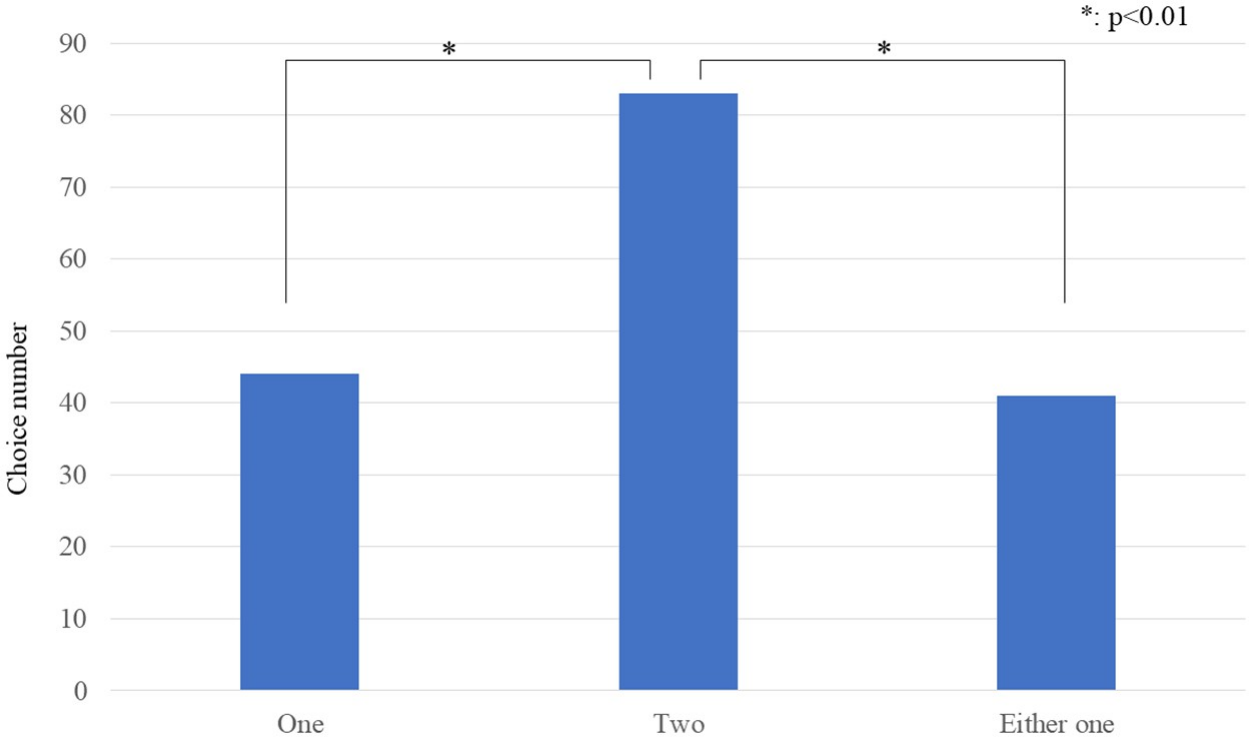
Preferecnce of the participants. Results of three-choice questions about the preferred number.

Fig 3 shows the number of each answer to the three-choice question. We conducted a chi-square test and identified a significant difference (*x*^2^(2) = 19.609, *p*<0.01). Multiple comparisons with Ryan’s tests [49] revealed that the participants significantly preferred *two* robots more than the other candidates (one < two, *p* < 0.01 and no difference < two, *p* < 0.01).

### Questionnaire results

**Fig 4:**
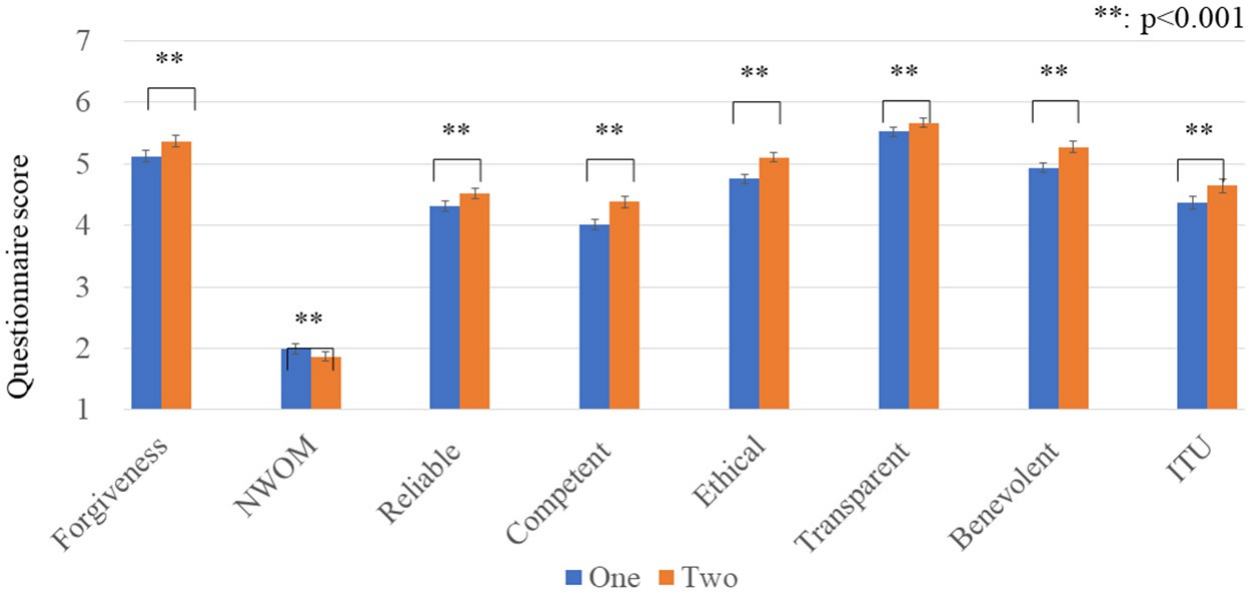
Average and S.E. of questionnaire results of Experiment I. The *two*-apologies condition is better than the *one*-apology condition from all questionnaire items.

Fig 4 shows the questionnaire results’ averages and standard error (S.E.). We conducted a paired-t test for each result, and all showed significant differences (forgiveness: *t*(1,167) = 4.034, *p <* 0.001, *d* = 0.778, NWOM: *t*(1,167) = 2.830, *p* = 0.005, *d* = 0.563, the reliable subscale; *t*(1,167) = 3.630, *p <* 0.001, *d* = 0.728, the competent subscale: *t*(1,167) = 6.115, *p <* 0.001, *d* = 0.785, the ethical subscale: *t*(1,167) = 6.757, *p <* 0.001, *d* = 0.665, the transparent subscale: *t*(1,167) = 2.585, *p* = 0.011, *d* = 0.716, the benevolent subscale: *t*(1,167) = 6.714, *p <* 0.001, *d* = 0.652, ITU: *t*(1,167) = 4.201, *p <* 0.001, *d* = 0.826). Thus, prediction 1 was supported.

### Free-description analysis

We gathered 287 sentences from the free-description forms. A coder categorized all the scripts as speech contents, bowing motions, additional behavior, and others (e.g., "no comments"). 37 sentences suggested adding speech content about the customers in the speech contents category, such as "Did anything get spilled on your clothes?" The remaining 45 sentences are just impressions of the contents and the tones of the robot’s voices. In the bowing category, 62 sentences suggested bowing more deeply, 39 suggested bowing longer, and nine suggested more bowing. In the additional behavior category, 40 sentences suggested wiping up the spill, 12 suggested that the robots perform separate roles, 10 suggested summoning a human clerk, and six suggested a replacement item instead of an apology. The other category had 27 sentences, although they did not suggest specific behaviors. We modified the robot behaviors in Experiment II based on the above information. The details are described in the next section.

## Discussion

### Potential benefits of apologies from multiple robots

Past studies reported the importance and effectiveness of apology behaviors of service robots [22–29]. However, these studies mainly focused on an apology from one robot, i.e., less focused on the effects of apologies from multiple robots, although using multiple robots could influence peoples’ perceptions strongly more than using one robot [30–34].

Our experiment results showed that the participants preferred apologies from two robots over one robot. In addition, all the questionnaire results showed they had positive impressions of two apologies: one from each robot. Higher forgiveness/ITU and lower NWOM scores indicate the robot’s apologies effectively mitigated the impressions of dissatisfaction caused by the robot’s mistake. Moreover, using multiple robots for apologies increased the trust scores of the robots and contributed to building a positive impression of the shop where the robots work. In particular, apologies from multiple robots were effectively perceived as more competent/ethical/benevolent perspectives from the viewpoint of the effect size. Therefore, these results answered research question 1: people preferred apologies from two robots more than from just one.

Even though multiple robots are effective for making an apology, preparing a sub-robot that only apologizes is inefficient. Each robot should have different roles, e.g., carrying a plate or taking orders at a restaurant. This result suggests that apologizing in support of another robot is a useful task for such robots.

### Additional apology behavior design

Although our experiment results supported prediction 1, the participants provided several suggestions on their free-description forms and possible actions for the sub-robot: cleaning up the mess and performing clearly separate roles. Investigating an appropriate behavior design for the sub-robot might increase the possible benefits of having multiple robots in daily situations.

Our participants also provided suggestions to modify the apologizing behaviors, such as speech content and bowing behaviors. We modified the robot’s behaviors in Experiment II based on them. Note that we rejected the behavior that summons a human clerk because her behaviors and characteristics would influence the apologies.

## Experiment II

Experiment II investigated the effects of the sub-robot and its behaviors, which were designed based on the free descriptions gathered from Experiment I.

### Hypothesis and prediction

The results of Experiment I showed the effectiveness of apologies from two robots over one robot and suggested the importance of investigating different role effects for the sub-robot. The participant opinions from the free-description forms include designing a sub-robot that cleans the spill and performs a separate role. As suggested by the participants, such an additional role for the sub-robot might effectively mitigate the failure of the main robot. On the other hand, we must determine whether the sub-robot should apologize with the main robot before cleaning up or should it only clean up the spill. We hypothesized that the sub-robot’s additional action would improve perceived impressions. Based on this hypothesis, we made the following prediction:

**Prediction 2**: If the sub-robot apologizes and cleans up during the main robot’s apology, people will accept the apologies and have more positive impressions than the sub-robot that only performs one action.

### Visual stimuli and conditions

Based on the results from Experiment I, we prepared three different videos. Two robots appeared in all the videos, but the sub-robot’s behaviors are different depending on the conditions. The main robot’s apologizing behaviors are based on the results of Experiment I: deeper and longer bows and verbally apologizing to its customers.

**Fig 5:**
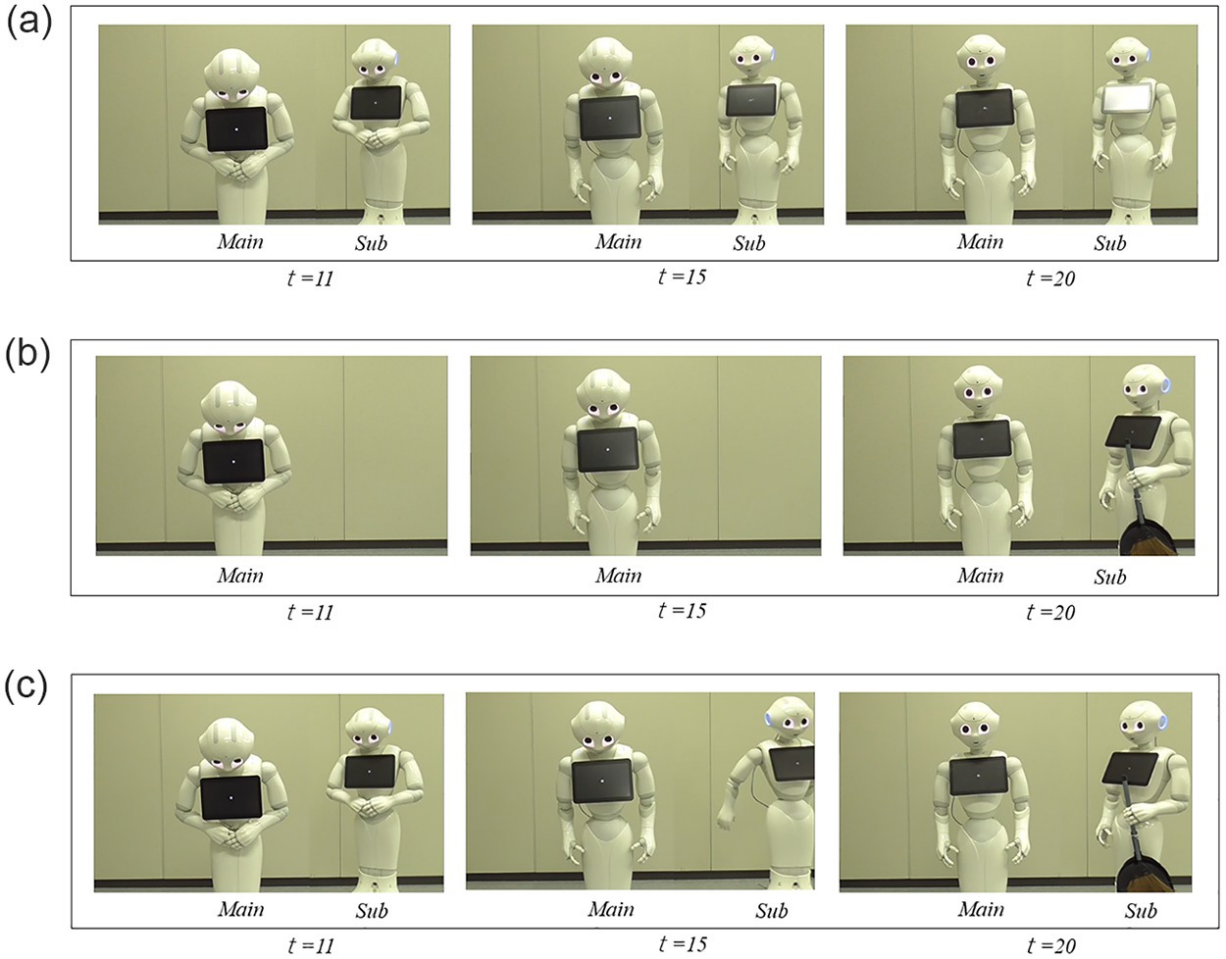
Three conditions. Differences among them are sub-robot’s role (*t* = seconds). (a) *Apologize-only* condition: sub-robot apologizes. (b) *Cleaning up-only* condition: sub-robot brings a broom and dustpan. (c) *Mixed* condition: sub-robot apologizes and returns with a broom and dustpan.

The first condition is *apologize-only* (Fig 5a). Here, similar to the *two* apologies condition in Experiment I, only the sub-robot apologizes. The second condition is *cleaning-up-only* (Fig 5b); after the main robot’s apology, the sub-robot enters from the right with a broom and a dustpan. In other words, we clearly separated the robot roles into apologizing and cleaning up. The third condition combines both into a *mixed* condition (Fig 5c); the sub-robot first apologizes, as in the *apologize-only* condition, moves to the right, and returns with a broom and a dustpan. Its timings in the *apologize-only* and *cleaning up-only* conditions are different because they are based on the *mixed* condition’s timing. The details of the timings of behaviors are described in Table 2.

**Table 2:** The timeline of the three conditions.

t (s)	Main robot	Sub robot (Apologize)	Sub robot (Cleaning)	Sub robot (Mixed)
1	Deliver an ice cream			
3	Drop the ice cream			
8		Enter from the right		Enter from the right
9	Apologize and bow			
10		Bow		Bow
14	Offers to serve new ice cream	Bow again		Exit from the right
15	Bow again			
17	Tell that no payment was required			
19			Enter from the right	Enter from the right
			with a broom/dustpan	with a broom/dustpan

### Measurement

We measured the same questionnaire items to investigate the effects of the robot apologies from Experiment I: the forgiveness scale [44], NWOM [44], the performance trust from the multi-dimensional measure of trust [45], moral trust from the multi-dimensional measure of trust [45], and intention to use (ITU) [46]. We excluded the three-choice question and the free-description forms in this experiment. Similar to Experiment I, we investigated the forgiveness, NWOM, and ITU scales about a shop and the trust scales toward the robot clerk serving the ice cream.

### Procedure

Similar to Experiment I, all the procedures were approved by the Advanced Telecommunication Research Review Boards (20-501-3). First, the participants read explanations of the experiment and how to evaluate each video, and then we verified that they could clearly hear the video’s audio. Next they observed one of the three candidates, completed the questionnaires, and watched all three videos. The order of the videos was counterbalanced. Finally, they answered dummy questions to determine how carefully they watched the video and to verify the quality of their answers because past research reported the need for such screening of participants in web surveys [47, 48].

### Participants

The experiment was conducted using the participant pools of the same Japanese survey company from Experiment 1. No participants in Experiment 1 were involved in Experiment 2; 609 people (337 women, 266 men, and 6 who declined to specify gender) joined. The screening process winnowed that number to 430 valid participants: 237 women, 188 men, and 5 who declined to specify.

## Results

### Questionnaire results

**Fig 6:**
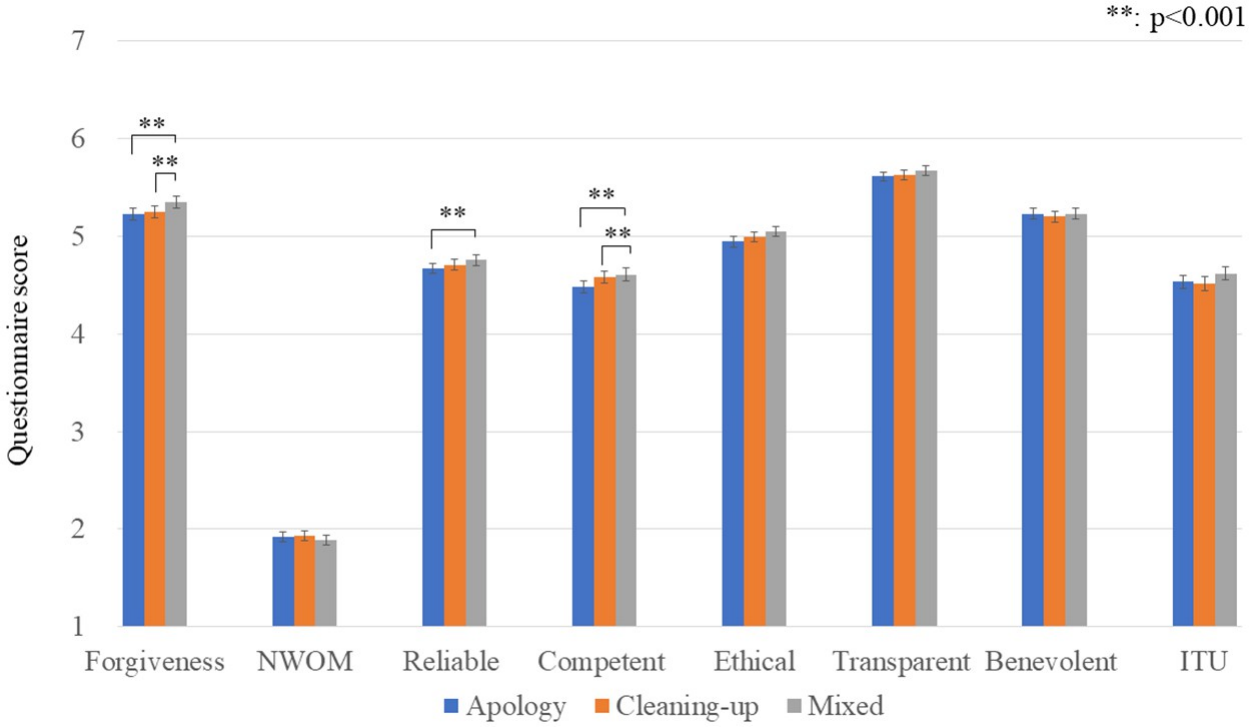
Average and S.E. of questionnaire results of Experiment II. The *mixed* condition is better than *apology* and *cleaning-up* conditions in forgiveness and competent scales.

Fig 6 shows the questionnaire results. We conducted a repeated ANOVA for each result, and only three scales showed significant differences: (forgiveness: *F*(2,858) = 5.536, *p* = 0.004, *partial η2* = 0.013, the reliable subscale; *F*(2,858) = 3.401, *p* = 0.034, *partial η2* = 0.008, the competence subscale: *F*(2, 858) = 5.096, *p* = 0.006, *partial η2* = 0.012). Multiple comparison with the Bonferroni method for forgiveness showed significant differences between conditions: *apology-only* < *mixed* (*p* = 0.011) and *cleaning-up-only* < *mixed* (*p* = 0.023). Multiple comparisons with the Bonferroni method for the reliable subscale showed significant differences between conditions: *apology-only* < *mixed* (*p* = 0.033). Multiple comparison with it for the competent subscale showed significant differences between conditions: *apology-only* < *mixed* (*p* = 0.008) and *cleaning-up-only* < *mixed* (*p* = 0.040).

Other scales did not show significant differences (NWOM: *F*(2,858) = 1.143, *p* = 0.319, *partial η2* = 0.003, the ethical subscale: *F*(2,858) = 1.792, *p* = 0.167, *partial η2* = 0.004, the transparent subscale: *F*(2,858) = 1.931, *p* = 0.146, *partial η2* = 0.004, the benevolent subscale: *F*(2,858) = 0.814, *p* = 0.444, *partial η2* = 0.002, ITU: *F*(2,858) = 2.875, *p* = 0.057, *partial η2* = 0.007). Thus, prediction 2 was partially supported.

## Discussion

### Potential benefits of apologizing in multiple roles

Our first experiment showed that the effectiveness of the apologies from multiple robots was preferred more than one robot, but the behavior design of the additional robot was relatively simple. We thought that investigating different behavioral effects of the additional robots would contribute to mitigating failures, then we compared three different behaviors in this experiment.

Our experiment results showed that participants preferred the *mixed* condition in the context of forgiveness, two of the trust viewpoints (reliable and competent) compared to the *apology* condition, and in the context of the competent trust viewpoint more than the *cleaning-up-only* condition. We believe that the differences in the competent scores are appropriate because multiple roles contributed to efficiently solving the problem. Thus, although the *mixed* condition did not show significant differences in the other measurements, apologies from two robots and separating their roles after they apologize is an appropriate strategy to mitigate the consequences of a robot’s mistakes. These results showed that having multiple roles enables the sub-robot to mitigate another robot’s mistake more efficiently. Similar to the results of Experiment 1, having multiple apologizing roles has merit for the shop where the robots are working.

The *cleaning-up-only* condition did not show any significant differences compared to the *apology* condition, although it did show some disadvantages compared to the *mixed* condition. Although the participants in Experiment 1 suggested cleaning up and separating the roles of the robots, they were ineffective unless combined with an apology. In the *mixed* condition, the participants had slightly more sincere and efficient impressions toward the robots compared to the other conditions where the sub-robot only did one task. we thought that the weak effects of *cleaning-up-only* conditions are mainly two kinds. The first one is the modified behaviors of the main robot; most of the questionnaire results of the *apologize-only* condition in Experiment 2 are better than the results of the two condition in Experiment I. The second one is that the suggested behaviors from the participants in Experiment 1 are less effective, although many of them thought such behaviors would be appropriate.

## General discussion

Since completely eliminating mistakes by service robots in actual environments is nearly impossible, effectively mitigating strategies is important. This study provided several implications about such strategies for robot mistakes. We thought that an apology from multiple robots might increase the perceived costs related to acceptance. Although quantifying the literal cost of increasing the number of robots is difficult, our experiment results showed that an apology from multiple robots is more acceptable. Here, we discuss implications and limitations based on two experiment results.

### Implications for apologies from multiple robots

Why are two robots better than one robot in an apology situation? One possible reason is that the sub-robot’s apology resembles a helping behavior toward the main robot, not only an apologizing behavior toward the customer. Past studies reported that helping or generosity is driven by receiving or observing assistance [50, 51]. If the participants thought that the sub-robot was both apologizing and supporting the main robot, such help-observation effects might have influenced their perceived impressions. This viewpoint seems related to increasing trust scores; people may more highly evaluate a robot supported by another robot than a robot without any support.

Another possible reason is a perceived apology cost. Simply using two robots incurs a higher cost than just using one. Related to this point, the participant might perceive the apology to be sincerer when two robots apologize. A past human-robot apology study also reported the effectiveness of a robot’s sincere apology [21–25]. These studies expressed sincerity by a single robot’s behaviors, and using multiple robots should increase the perception of such sincere impressions. In addition, we should consider a peer pressure effect from multiple robots. People’s decision-making is influenced by the opinions of others [52, 53]. Several past studies reported conformity effects from robot groups, which change people’s behaviors and impressions [54, 55]. Although the number of robots in our study is just two (not many robots), the participants might have felt peer pressure from the robots.

Based on these considerations, one remaining unknown point is the most effective number of robots. Perhaps simply increasing the number of robots is not effective for acceptable apology behavior because people might become suspicious and cynical toward so many apologizing robots. Since past studies with multiple robots for information-providing situations focused less on an effective number of social robots, this issue remains unresolved. Another study that focused on peer pressure effects from social robots investigated the effective number and reported that six robots strengthened the peer pressure effects more than two or four robots [55], although apologies from six robots is excessive. In fact, a few participants in Experiment I described that a scenario where more robots apologized might create negative impressions. Investigating the best balance between the number of apologizing robots and the perceived cost is interesting future work.

Another implication is the robot’s appearance. In this study, we used Pepper as a humanoid robot that can serve items. However, if the robot’s appearance is more humanlike, people’s perceptions will be different. Using different robots also influences forgiveness; if the sub-robot is more human-like or has more authority, people will accept its apology more. On the other hand, if the sub-robot seems to have less authority, people’s perceptions will also be different. Using android robots with a human-like appearance [56, 57] might provide additional knowledge for robot-apology behavior designs.

### Implications for apologies by robots and people

Until robot clerks are sufficiently diffused into human society, human and robot clerks might work together. Although this study did not investigate the effects of apologies due to metaphysical differences, we believe that a human clerk’s apology will be more effective than one from a robot clerk. A past study reported the importance of apology cost toward acceptance [31], and human clerks currently have higher social status than robot clerks.

We believe that investigating the effects of additional apologies by robots with human mistakes will be fruitful, e.g., a human clerk’s mistake, and then a robot clerk apologizing with the human clerk. In addition, the effectiveness of a robot’s apology will be influenced by relationships and such roles as a child/childcare robot, senior citizen/nursing robot, student/teacher robot, etc.

### Limitations

This study has several limitations, including using specific robots, situations, and context. Therefore, as described above, using different robots, environments, and contexts, such as the responsibility of failure is not lying to the robot clerk (e.g., an accident caused by other people), would have different results with multiple robots’ apologies. It investigated the perceived impressions toward a robot’s failure by videos, not actual failures in daily situations. In fact, related studies in behavioral economics are performed with real money (typically in the trust game, e.g., [58, 59]). Therefore, conducting additional studies in actual settings is critical to investigate the effects of multiple robot apologies. On the other hand, conducting real situations under such experiment settings is complicated by ethical viewpoints. Therefore, several related studies in human-robot interaction conducted a video-based survey to investigate the effects of robot behaviors in specific situations with difficulties that reproduced real situations [25, 60]. We used a video-based survey approach to provide insight into designing a robot’s behaviors in specific situations, including mitigating failures by robots.

Since we only conducted our experiments with Japanese participants, we must consider generality and cultural differences [15, 61, 62]. One future work will investigate the effectiveness of using multiple robots to mitigate failures in different cultures. Related to such future work, investigating the influence of different kinds of robots and behaviors, e.g., more humanlike robots, is essential for understanding the relationships between cultural differences and acceptable apologies by robots. In addition, different environments such as high-end restaurants will undoubtedly also influence the perceived impressions of robot failures.

## Conclusion

This study investigated the effects of apologies by multiple robots to mitigate the mistakes made by robots. We first prepared different visual stimuli where one or two robots apologized in a restaurant environment. The main robot makes a mistake and apologizes, and then the sub-robot apologizes with the main robot. We conducted a web-survey-based experiment to investigate the perceived impressions whose results showed that apologies from two robots are preferred over an apology from just one. We also gathered suggestions from participants about modifying the sub-robot’s behaviors while the main robot apologizes. Based on such free descriptions, we created three different behaviors for the sub-robot: apologize-only, cleaning-up-only, and mixed combinations. We conducted another web-survey-based experiment to investigate the perceived impressions and identified a part of the advantages of both combinations over apologizing-only and cleaning-up-only. Our results suggest the importance of supporting a robot that makes a mistake with another robot that can apologize with it, and provide the evidences about the effectiveness of using multiple robot apologies.

## Supporting information

S1 File Anonymized data set and questionnaire items. (XLSX)
